# The Reformatory Treatment of Inebriates

**Published:** 1905-07-29

**Authors:** 


### THE REFORMATORY TREATMENT OF INEBRIATES.

Mr. R. W. Branthwaite, in reporting on the working of the Inebriates Acts, dwells on the importance of sentencing inebriates for a long period early in their careers. He contrasts the cases of two women, one of whom has served 180 terms of imprisonment for drunkenness during the last 25 years, besides being charged 40 more times for offences more or less directly due to her drunken habits. When at last she was sent for two years to an inebriate reformatory, she was too degraded to profit by the treatment, and of the 24 months which have elapsed since her discharge, she has spent 18 in prison. Against this case Mr. Branthwaite sets that of a married woman who after only one year of police-court history, had the good fortune to be arraigned before a magistrate, who, as Mr. Branthwaite, says, "understood the advantage of striking hard and striking early." This gentleman sentenced her to three years' detention in a reformatory, the result of which was so satisfactory that since she went out on license in February 1902, she has not touched liquor, and all reports concerning her behaviour and the condition of her home are excellent. Although these two cases may present the extremes of success and failure, most people know that the best, if not the only, hope of reforming the inebriate lies in putting him or her out of temptation for a prolonged peric[illegible] as early as possible, and while in the intervals of the [illegible]ng there is a feeling of shame at the consciousness of fall. [illegible]t sentences are useless, if not worse. As a rule the pris[illegible] returns to the world with a more violent craving for indulgen[illegible] than ever. It is said that there is a colonial magistrate who has a short way with offenders of all classes. He adds together the sum of all previous convictions against a prisoner, and sentences him for the period thus indicated. On this principle a regular offender would soon receive a sentence sufficiently long for any reformative influences by which he can be affected to have time to work. This method approximates to the indeterminate sentence of which so much has been heard in England, but, unfortunately, so little done. It may be called the cumulative sentence, and, whether applicable to other offences or no, is well adapted to dealing with cases of drunkenness, where the craving for the indulgence also is cumulative. The provisions of the Inebriates Acts are framed to prevent too severe a sentence being passed on any who are guilty of only an exceptional or even occasional fall, and, when a case appears at the police-court with sufficient frequency to permit of a long sentence, magistrates would be well advised to believe that it is the truest kindness to "strike hard and strike early."

